# 30 years of fatal dengue cases in Brazil: a review

**DOI:** 10.1186/s12889-019-6641-4

**Published:** 2019-03-21

**Authors:** Priscila Conrado Guerra Nunes, Regina Paiva Daumas, Juan Camilo Sánchez-Arcila, Rita Maria Ribeiro Nogueira, Marco Aurélio Pereira Horta, Flávia Barreto dos Santos

**Affiliations:** 1 Viral Immunology Laboratory, Oswaldo Cruz Institute, IOC, Oswaldo Cruz Foundation, FIOCRUZ, Avenida Brasil, 4365. Manguinhos, Rio de Janeiro, Brazil; 2 Clinical Epidemiology Laboratory, Evandro Chagas Clinical Research Institute-FIOCRUZ, Avenida Brasil, 4365. Manguinhos, Rio de Janeiro, Brazil; 3 Flavivirus Laboratory (LABFLA), Oswaldo Cruz Institute – FIOCRUZ, Avenida Brasil, 4365. Manguinhos, Rio de Janeiro, Brazil

**Keywords:** Dengue mortality, Surveillance, 30 years, Brazil

## Abstract

**Background:**

Over the last 30 years, extensive dengue epidemics have occurred in Brazil, characterized by emergences and re-emergences of different serotypes, a change in the epidemiological profile and an increase in the number of severe and fatal cases. Here, we present a review on the dengue fatal cases that occurred in Brazil in 30 years (1986–2015).

**Methods:**

We performed an ecological study by using secondary data on dengue fatal cases obtained in the National System of Reported Diseases (Sistema de Informação de Agravos de Notificação -SINAN) and in the Mortality Information System (SIM), both maintained by the Brazilian Ministry of Health. Cases were analyzed by region, demographic variables, clinical classification and complications based on the data available.

**Results:**

In 30 years (1986–2015), the Southeast region reported 43% (*n* = 2225) of all dengue deaths in the country. The Midwest region was responsible for 18% of the fatal cases. After 2000, deaths occurred in almost all states, with the exception of Santa Catarina and Rio Grande do Sul, South region. From 2006 to 2010, the number of deaths increased, with higher rates of mortality, especially in Goiás and Mato Grosso. From 2011 to 2015, Goiás became the state with the highest mortality rate in the country, and Rio Grande do Sul reported its first dengue deaths. In 30 years, a total of 2682 dengue deaths occurred in males and 2455 in females, and an equal distribution between the sexes was observed. From 1986 to 2006, dengue deaths occurred predominantly in individuals over 15 years old, but this scenario changed in 2007–2008. After 2009, fatal cases on individuals above 15 years old became more frequent, with peaks in the years of 2010, 2013 and 2015.

**Conclusions:**

The Brazil is experiencing a hyperendemic scenario, which has resulted in the co-circulation of the four DENV serotypes and with the increasing occurrence of severe and fatal cases. The disease surveillance and studies characterizing what has been reported overtime, are still important tools to better understand the factors involved in the disease outcome.

## Background

Dengue viruses (DENV) are arboviruses belonging to the *Flaviviridae* family and the genus *Flavivirus*, and are represented by four antigenically distinct serotypes (DENV-1 to 4) causing a mild self-limiting illness or more severe forms of the disease and death [1]. According to WHO [1], currently dengue cases can be classified as dengue without warning signs, dengue with warning signs and severe dengue. A severe dengue case is characterized by severe bleeding, severe organ involvement and severe plasma leakage. The viruses are responsible for high rates of disease and mortality [2]. Dengue is a mosquito-borne viral disease endemic in several tropical and sub-tropical countries worldwide and, in recent decades the disease has grown drastically throughout the world [3]. Globally, it is estimated an average of 9 thousand dengue deaths per year from 1990 to 2013 have occurred [4]. In the Americas, dengue has an endemo-epidemic pattern with outbreaks occurring every 3 to 5 years [5].

From 1995 to 2015, more than 18 million cases of dengue were reported throughout the American continent and, about 14 million cases were reported only in South American countries. Brazil contributed 55% of the cases reported in the Americas over this period. A total of 8788 fatal cases were confirmed in the Americas, and Brazil accounted for 48% of those cases [6]. Despite that, dengue cases are still underreported and many cases are incorrectly classified, with one notification for every twenty cases of dengue fever (95%) [7, 8].

Over the last 30 years, extensive dengue epidemics have occurred in Brazil, characterized by the emergence and re-emergence of different serotypes, a change in the epidemiological profile and an increase in the number of severe and fatal cases. Here, our goal is to present a review on the fatal dengue cases that occurred in Brazil over 30 years (1986–2015) based on the Brazilian Dengue Surveillance Systems, as understanding the patterns of case fatalities, may be critical for dengue case management in the country.

## Methods

We performed an ecological study by using secondary data from the dengue epidemics available in Brazil. Official data on dengue fatal cases occurred from 1986 to 2013, from TabNet (DATASUS) from the National System of Reported Diseases (Sistema de Informação de Agravos de Notificação -SINAN) and from the Mortality Information System (SIM), both maintained by the Brazilian Ministry of Health (MoH), were obtained. Cases occurring in 2014 and 2015 were obtained from epidemiological reports available at http://portalms.saude.gov.br/boletins-epidemiologicos.

Dengue severity was considered according to the final classification of the Brazilian MoH and to the epidemiological reports available, as follows: Dengue with complications (DCC), Dengue Hemorrhagic Fever (DHF), Dengue Shock Syndrome (DSS) and Severe Dengue (SD). In this study, the 1997 World Health Organization (WHO) dengue case classification (DHF and DSS) was used from 1986 to 2000. From 2000 to 2013, the Brazilian MoH DCC classification was used to define severe dengue cases that did not meet the WHO criteria for DHF/DSS and, from 2014 and on, the 2009 WHO dengue case classification, Dengue with warning signs (DwWS) and SD were employed [1–6, 9]. Here, we considered dengue deaths to be reported in the SINAN database filled out as “death due to dengue,” or from the SIM database where cause of death was with the code “A90” or “A91,” according to the 10th International Classification of Diseases (ICD-10).

The case fatality rate of each classification was calculated using number of deaths from DHF/DSS, DCC, DwWS or SD per number of confirmed cases from each classification × 100. The overall fatality rate was calculated by the sum of each classification per number of dengue confirmed cases × 100. The mortality rate was calculated using the number of deaths per dengue per total number of the locality’s population, per year × 100,000 inhabitants. The population data of each year and by region were obtained from Instituto Brasileiro de Geografia e Estatística (IBGE) available at https://www.ibge.gov.br. Cases were analyzed by region, demographic variables and clinical classification based on the data available on the reporting and investigation forms using a database in Excel Software. The maps were made using the TerraView 4.2.2 (INPE, SP, Brazil) software.

Odds ratio (OR) of dengue fatal cases occurred in Brazil from 1987 to 2015 was calculated with a 95% confidence interval (CI) and *p*-values for each year, with 1986 as the reference year. The analysis was performed by GraphPad Prism software version 6. We used 1986 as the reference year as it was the first year of dengue introduction in Brazil and the first year of data availability on the Brazilian MoH database. All data are available from the Brazilian MoH and do not need permission for access.

## Results

### Overview on dengue epidemics in Brazil

The first reports of a disease with signs and symptoms compatible with dengue fever in Brazil, date back to 1846 [10]. Late 1981 and early 1982, a first dengue outbreak, caused by DENV-1 and DENV-4 was characterized in Brazil, which was restricted to the city of Boa Vista, Roraima (RR) in the north region [11]. In1986, after 4 years without dengue cases confirmation, an epidemic occurred due to the DENV-1 introduction in the state of Rio de Janeiro (RJ), which spread to other states [12]. Five fatal cases were confirmed in 1986. DENV-2 was detected, for the first time in RJ, in 1990 [13], when the first DHF/SCD cases occurred (*n* = 8). In the following years, DENV-1 and DENV-2 co-circulated and caused epidemics throughout the country [14] . Through 1999, a total of 75 fatal cases were reported (1991–1999), Fig. 1.

**Fig. 1:**
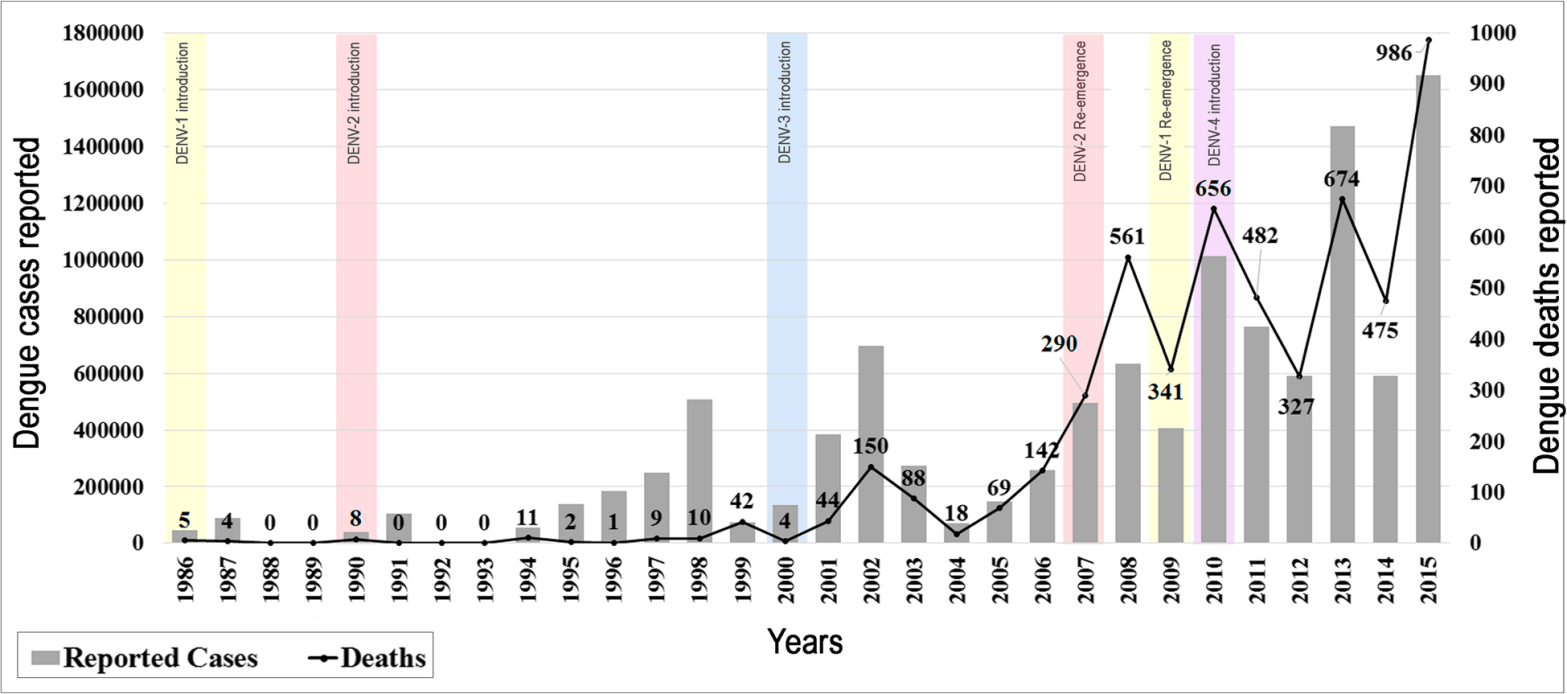
Dengue cases and dengue fatal cases reported in Brazil in 30 years (1986 to 2015). The bars show the number of dengue cases reported. The numbers of deaths are shown in lines and *y* axis to the right. The colored squares demonstrate the introduction and re-emergence of the distinct dengue serotypes

In December 2000, a newly introduced serotype, DENV-3, was initially detected in RJ [15] and quickly spread to other states of the country. The 2002 epidemic, caused mainly by DENV-3, was the largest and most severe epidemic experienced in the country so far, with increased hospitalizations and 150 deaths confirmed. It was suggested that, the introduction of a new serotype of Asian origin (Genotype III), would have been an explanation for the severity of the epidemic [16]. Despite the prevalence of Genotype III in Brazil, the co-circulation of Genotypes III and V was detected in Rondônia [17]. In 2002, the number of DHF deaths exceeded malaria deaths for the first time in the country [14].

Seventeen years after its introduction, DENV-2 reemerged in 2007 causing a major epidemic in 2008, with a higher proportion of DHF, more than double the number of cases reported in previous years [18]. A total of 561 deaths, mainly caused by this serotype, were reported only in that year (Fig. 1A). Oliveira et al. [19] observed that the DENV-2 emerged in the 2008 epidemic was genetically different from the strain introduced in 1990, and despite belonging to the same genotype, those viruses were considered a new lineage (Lineage II). Studies by Faria et al. [20] concluded that there were no nucleotide changes between the two strains that led to an increase in the severity of Lineage II viruses. On the other hand, Nunes et al. [21] demonstrated that the viremia of the DENV- 2 Lineage I cases was lower than that observed by Lineage II cases. Furthermore, severe cases caused by Lineage II, had 1000 times more circulating virus than those from Lineage I. The factors that led to the severity of this particular epidemic are still unclear. However, one cannot exclude the difficulties experienced in public health systems in controlling the epidemic, and the situation caused panic and insecurity throughout the Brazilian society [22, 23].

In 2009, DENV-1 reemerged with the possibility of a new epidemic, considering the low circulation of this serotype since the beginning of the decade. The 2010 epidemic presented a pattern quite different from the 2002 and 2008 epidemics, with the highest number of deaths (*n* = 656), reported. The DENV-1 isolated in Brazil between 2009 and 2010, belonged to Genotype V (American /African) and grouped in a clade (Lineage II) distinct from that of the previous isolates (Lineage I). Moreover, strains isolated in 2011 grouped in another distinct clade (Lineage III) [24]. The introduction of new strains resulted in the substitution of the circulating lineage and the increase in the genetic diversity of DENV-1, probably as a result of local evolution, or introduction of exogenous viruses during the same period or at different times [25].

In 2010, the risk of DENV-4 reintroduction into the country was imminent, as this serotype circulated in neighboring countries such as Venezuela and Colombia [26]. However, only in July of 2010, the first DENV-4 cases were identified in RR and Amazonas (AM), about 30 years after its first detection in the country. Less than 20 cases of DENV-4 were confirmed during the second half of 2010, and the first cases resulting from the spread of the virus, were detected only in January 2011, isolated in Amazonas and Pará. In March of 2011, the first DENV-4 cases were reported in RJ, introduced by the municipality of Niteroi [27, 28].

An increase in deaths was evidenced, especially in 2015, with an explosive epidemic of 1,649,008 dengue cases reported and 986 fatal cases confirmed. In 30 years a total of 11,084,755 suspected dengue cases were reported, with the confirmation of 5399 deaths nationwide (Fig. 1). The years that had the greatest chances of death were 2007–2009 (CI 95% 2.23–19.8), mainly due to the DENV-2 epidemic. The years of 2014–2015 had OR of 3.08–17.97 and, despite the co-circulation of the four serotypes, DENV-4 was predominant. In 2010–2011 OR were (CI 95% 2.492–14.48), when DENV-1 reemerged and DENV-4 was introduced (Table 1).

**Table 1:** Odds Ratio of dengue fatal cases occurred in Brazil from 1987 to 2015, considering the first epidemic year (1986)

Year	Reported cases	Deaths	OR over 1986	Confidence interval	*P* value
1986	46,309	5	–	–	–
1987	88,407	4	0.419	0.11–1.56	*0.2903*
1988	1570	0	0.000	0–32.02	*> 0.999*
1989	5367	0	0.000	0–9.36	*> 0.999*
1990	40,279	8	1.840	0.60–5	*0.4054*
1991	104,399	0	0.040	0–0.73	*0.0027*
1992	1696	0	0.000	0–29.64	*> 0.999*
1993	7374	0	0.000	0–6.81	*> 0.999*
1994	56,691	11	1.797	0.62–5.17	*0.3219*
1995	137,308	2	0.135	0.02–0.69	*0.0134*
1996	183,762	1	0.050	0–0.43	*0.0017*
1997	249,239	9	0.334	0.11–0.99	*0.055*
1998	507,715	10	0.182	0.06–0.53	*0.006*
1999	74,670	42	5.210	2.06–13.17	*< 0.0001*
2000	135,228	4	0.274	0.07–1.02	*0.053*
2001	385,783	44	1.056	0.42–2.66	*> 0.999*
2002	696,472	150	1.995	0.82–4.86	*0.1352*
2003	274,975	88	2.964	1.20–7.29	*0.0111*
2004	70,174	18	2.376	0.88–6.39	*0.0896*
2005	147,039	69	4.346	1.75–10.77	*0.0002*
2006	258,680	142	5.084	2.08–12.40	*< 0.0001*
2007	496,923	290	5.405	2.23–13.08	*< 0.0001*
2008	632,680	561	8.212	3.40–19.81	*< 0.0001*
2009	406,269	341	7.774	3.21–18.80	*< 0.0001*
2010	1,011,548	656	6.006	2.49–14.48	*< 0.0001*
2011	764,032	482	5.843	2.42–14.10	*< 0.0001*
2012	589,591	327	5.137	2.12–12.42	*< 0.0001*
2013	1,470,487	674	4.245	1.76–10.23	*0.0007*
2014	591,080	475	7.443	3.08–17.97	*< 0.0001*
2015	1,649,008	986	5.538	2.30–13.34	*< 0.0001*

Footnote: To compare the Odds Ratio of deaths occurred from 1987 to 2015, we calculated OR values, confidence intervals and P-values, setting the year of 1986 as the comparison year. Values were calculated using GraphPad Prism version 6 software

### Fatal dengue and regions

In 30 years, the Southeast region reported 43% (*n* = 2225) of all dengue deaths in the country. São Paulo (SP) confirmed 945 fatal cases, RJ, 738, Minas Gerais (MS) and Espírito Santo (ES) registered 430 and 196 deaths, respectively. In the Northeast, the states with the highest number of fatal cases were Ceará (CE) with 506, Pernambuco (PE) with 277, Bahia (BA) with 228 and Maranhão (MA) with 166. The Midwest region was responsible for 18% of the fatal cases, where the state of Goiás (GO) reported 600 deaths, Mato Grosso (MT), 187, Mato Grosso do Sul (MS), 128 and Distrito Federal (DF), 66. In the North region, only 7% of the deaths were confirmed. Pará (PA) was the state that reported the highest number of dengue deaths (*n* = 141) in the period. The South region, historically less affected by dengue cases, reported consequently the lowest number of dengue fatal cases (2%). Only Paraná (PR) (*n* = 108) and Rio Grande do Sul (RS) (*n* = 4) reported dengue deaths.

**Fig. 2:**
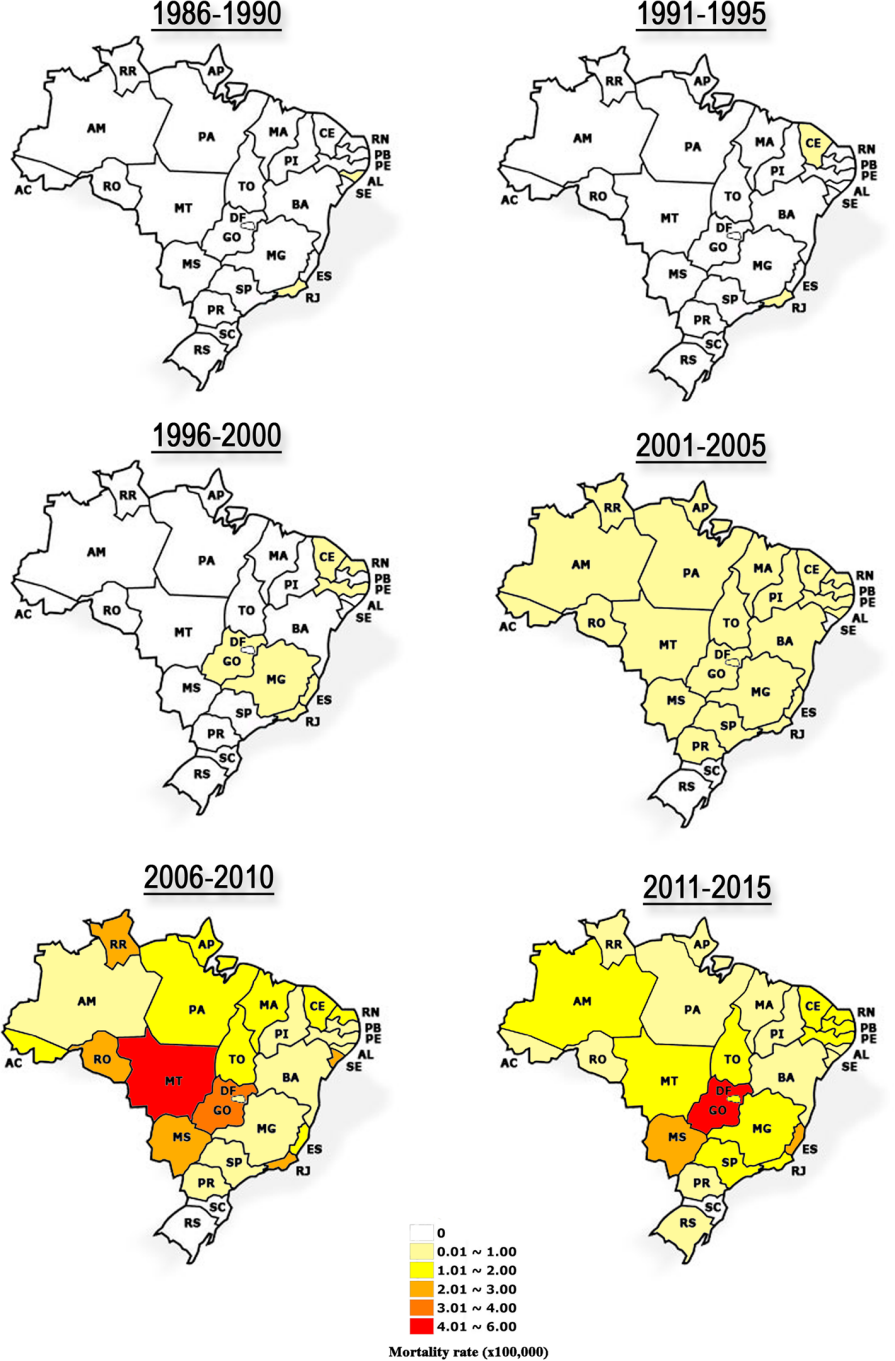
Five-year dengue mortality rate per state, Brazil, 1986–2015. Mortality rate per 100,000 populations. State abbreviations: Acre (AC); Alagoas (AL); Amapá (AP); Amazonas (AM); Bahia (BA); Ceará (CE); Distrito Federal (DF); Espírito Santo (ES); Goiás (GO); Maranhão (MA); Mato Grosso (MT); Mato Grosso do Sul (MS); Minas Gerais (MG); Pará (PA); Paraíba (PB); Paraná (PR); Pernambuco (PE); Piauí (PI); Roraima (RR); Rondônia (RO); Rio de Janeiro (RJ); Rio Grande do Norte (RN); Rio Grande do Sul (RS); Santa Catarina (SC); São Paulo (SP); Sergipe (SE); Tocantins (TO)

During 30 years of epidemics, we have observed that RJ historically contributed to the introduction and dissemination of DENV-1, 2 and 3, and since then, has constantly reported dengue fatal cases (Fig. 2). After 2000, deaths occurred in almost all states, with the exception of Santa Catarina (SC) and RS. From 2006 to 2010, possibly due to the introduction of DENV-3 and DENV-4, and re-emergence of DENV-1 and DENV-2, the number of deaths increased, with higher mortality rates in the states of RJ, Sergipe (SE), MS, Rondônia (RO) and RR. Rates were even higher in GO, but the state of MT had the highest mortality rate in this period. From 2011 to 2015, GO became the state with the highest mortality rate in the country, and RS reported the first dengue fatal outcomes.

The analysis of the mortality rates by municipality showed an increase of dengue fatal cases and distribution by the Brazilian territory over the years. In 2008, the North, Northeast and Southeast regions had higher mortality rates. In 2009, dengue deaths were distributed in the North and Midwest regions. From 2010 to 2012, dengue deaths occurred throughout the Brazilian regions (Fig. 2).

### Dengue classification

According to the WHO [9] dengue case criteria, infections were classified as dengue fever (DF), DHF and DSS. However, in 2000, the Brazilian MoH proposed the DCC classification, to define severe dengue cases that did not meet the WHO criteria for DHF/DSS. From January 2014 and on, Brazil adopted the new WHO 2009 classification. Therefore, in this analysis, DHF/DSS, DCC, DwWS and SD denominations were used, considering the epidemic year analyzed. The timeline and the characteristics of each classification are available in Table 2.

**Table 2:** Timeline and characteristics of dengue classifications used over 30 years of dengue fatal cases investigation in Brazil

Dengue classification	Source	Classifications	Years of use in Brazil
World Health Organization (WHO), 1997	World Health Organization (WHO), after a study based on dengue on children in Thailand in the 1950s and 1960s, with modifications in 1986 and 1997 [34].	DHF and DSS	From 1986 to 2000
Ministry of Health of Brazil, 2000	Brazilian Ministry of Health, used to define dengue severe cases that did not meet the WHO criteria for DHF / DSS. Used only in Brazil.	DCC	From 2000 to 2013
WHO, 2009	World Health Organization (WHO), based on the results of a multicenter study (DENCO) conducted in Southeast Asia and Latin America to assess the limitations of the 1997 classification.	DwWS and SD	From 2014 to present

DHF: Dengue haemorraghic fever, DSS: Dengue shock syndrome, DCC: Dengue with complications, DwWS: Dengue with warning signs, SD: Severe dengue

**Fig. 3:**
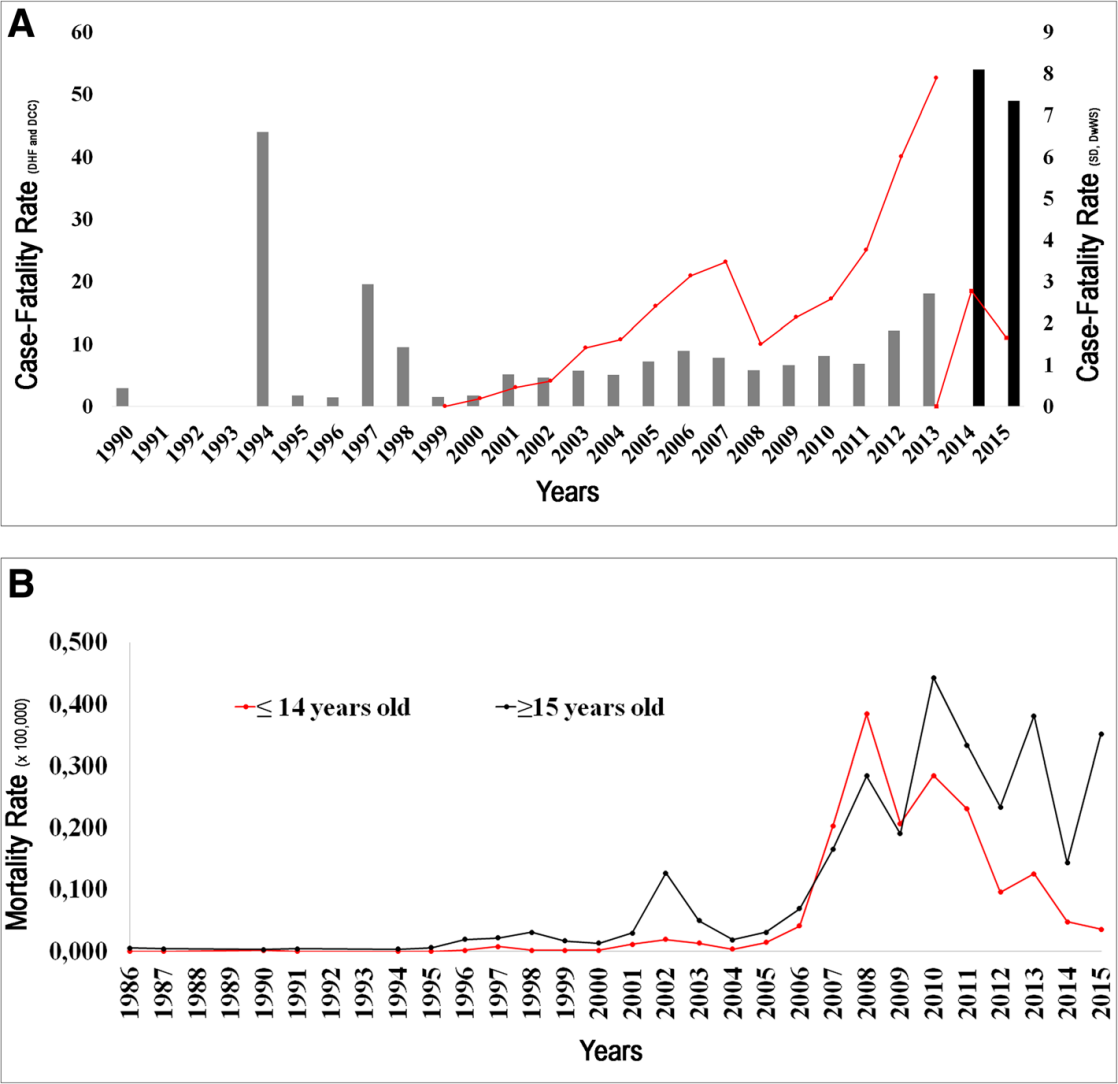
(**a**) 30-year dengue cases fatality rate by DHF, DCC, DwWS and SD and (**b**) distribution of mortality rates (per 100,000 populations) by age and year of occurrence, Brazil, 1986–2015. In Fig. A: Dengue case fatality rate is demonstrated in percentage (%). The bars show the fatality rate by the Dengue Hemorrhagic Fever (DHF) and Severe Dengue (SD) classifications. Dengue cases fatality rate with Dengue with Complications (DCC) and Dengue with Warning Signs (DwWS) are shown in lines. The axes *y* left are of the rates by the classification of DHF and DCC, whereas the axis *y* right are the values of the rates classified with SD and DwWs

DHF cases fatality rates were high in 1994, 1997, 1998, 2006, 2012, and 2013. By DCC, deaths were more frequently reported in 2003, 2006, 2007, with increasing numbers from 2008 to 2013 and the latter, being the highest peak of DCC mortality. Considering the new classification, 3% of DwWS patients died in 2014. In 2014 and 2015, 8 and 7% of SD cases died, respectively (Fig. 3A).

**Fig. 4:**
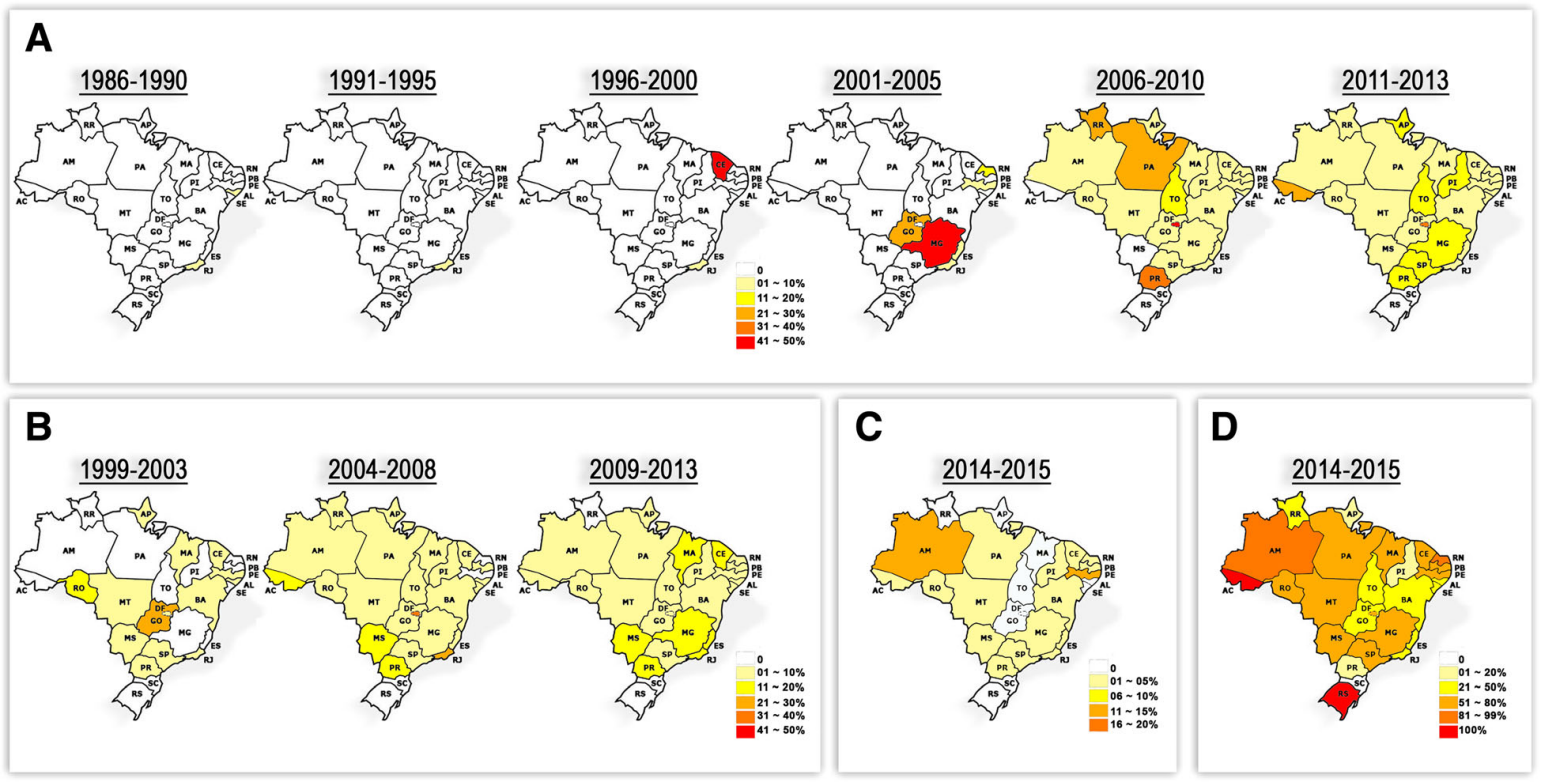
Five-year dengue case fatality rate per state from 1986 to 2015, Brazil. Case fatality rate by (**a**) DHF; (**b**) by DCC; (**c**) by DwWS and (**d**) by SD. Dengue case fatality rate is demonstrated in percentage (%). DHF: Dengue Hemorrhagic Fever; DCC: Dengue with Complications; DwWS: Dengue with Warning Signs; SD: Severe Dengue. State abbreviations: Acre (AC); Alagoas (AL); Amapá (AP); Amazonas (AM); Bahia (BA); Ceará (CE); Distrito Federal (DF); Espírito Santo (ES); Goiás (GO); Maranhão (MA); Mato Grosso (MT); Mato Grosso do Sul (MS); Minas Gerais (MG); Pará (PA); Paraíba (PB); Paraná (PR); Pernambuco (PE); Piauí (PI); Roraima (RR); Rondônia (RO); Rio de Janeiro (RJ); Rio Grande do Norte (RN); Rio Grande do Sul (RS); Santa Catarina (SC); São Paulo (SP); Sergipe (SE); Tocantins (TO)

The five-year case fatality rate of each state is shown in Fig. 4. From 1986 to 1990 the DHF fatality rate was up to 10% in RJ and Alagoas (AL). From 1991 to 1995, only RJ reported a DHF fatality rate up to 10%. That rate was five times higher (up to 50%) in CE, from 1996 to 2001. On the following years (2001 to 2005), fatality rates were up to 50% in MS, followed by GO (up to 40%), PB (up to 20%), and RJ, ES and PE with up to 10% of DHF cases evolving to death. From 2006 to 2010, this scenario changed and case fatality rate increased in almost all states, being higher in DF (up to 50%), PR (up to 40%), PA and RR (up to 30%) and TO (up to 20%). From 2011 to 2013, the states of PR, SP, MG, TO, PI, AP had up to 20% DHF fatality rate, however those were higher in CE with 21–30% and DF with 31–40% (Fig. 4A). In the years of 1999–2003, 11 to 20% of the cases with DCC died in RR, and in MT, MS, PR, SP, RJ, BA, SE, AL, CE, MA and AP, up to 10% (Fig. 4B).

From 2004 to 2008, increased numbers of fatal cases by DCC were also reported in other Brazilian states, and AC, MS, PR reported up to 20%, RJ up to 30% and DF up to 40% of fatality rates, Fig. 3B. In 2009 and 2013 the number of deaths was lower, and the states of MA, CE, MS, MG, RJ, ES and PR had up to 20% of case fatality rate. Only the state of SC did not report fatal cases by DCC (Fig. 4B).

Considering the new WHO [1] criteria, case fatality rate by DwWS in PA, AC, RO, MT, MS, PR, SP, MG, RJ, ES, BA, CE RN and PE was around 5%, however in PB and AM, it reached 15% in 2014–2015 (Fig. 4C). In the same period, deaths from SD occurred in all states, except in SC, with high case fatality rates in the states of AC and RS (100%), AM, PB and DF with 81 to 99%, MG, SP, MS, MT, MA, PE, PB, CE, PA, RO with 51 to 80%, RJ, ES, BA, GO, TO, RR, SE, AL with 21 to 51% and in PR, PE and AP, with up to 20%, Fig. 4D.

### Demographic variables associated to dengue fatal cases: sex and age

In the 30 years period (1986–2015), a total of 2682 dengue deaths occurred on males and 2455 on females and, during the years, an equal distribution between the sexes, was observed.

From 1986 to 2006, dengue deaths occurred more often in individuals over 15 years old, Fig. 4B. This changed in 2007–2008, with the DENV-2 re-emergence, as more than 53% of the dengue deaths cases occurred in children 15 years old and under. In 2008 alone, 190 fatal cases on children from that age group were reported (Fig. 3C). After 2009, there was a decrease in fatal cases in children 15 years old and under, while fatal cases on individuals above 15 years old became more frequent, with peaks in the years 2010, 2013 and 2015.

## Discussion

The consecutive introduction of distinct DENV serotypes overtime, resulted in a hyperendemic scenario, with the co-circulation of all serotypes and, an increase in deaths, was evidenced, especially in 2015. However, the years that had the greatest chances of death were between 2007 and 2009, mainly due to the DENV-2 epidemic.

As Brazil is the second largest and most populated country in the Americas, it is important to understand the contribution of the distinct regions in the occurrence of dengue deaths. Historically, the regions in the country with highest dengue incidences and fatal cases have been the Southeast, followed by the Northeast region. During 30 years of epidemics, RJ, in the Southeast region, has historically contributed to the introduction and dissemination of three of the four DENV serotypes (DENV-1 to 3), and since then, has constantly reported dengue fatal cases.

One well-characterized study by Paixão [29] analyzed the trends and factors associated with dengue mortality and fatality in Brazil from 2001 to 2011, and reported the results on the analysis of 3156 deaths. It was shown that the Southeast and Northeast regions accounted for more than 70% of fatal cases. Moreover, mortality rates increased during the period and that the factors associated with mortality were inequality, high income per capita and higher populations inhabiting urban areas [29].

According to the WHO [9] dengue case criteria, infections were classified as dengue fever (DF), DHF and DSS. However, due to difficulties in using this classification [30], mostly due to changes in the disease epidemiology, a new classification was needed. In 2000, the Brazilian MoH proposed the DCC classification, to define severe dengue cases that did not meet the WHO criteria for DHF/DSS [31, 32]. DCC was characterized when the dengue patient presented at least one of the following: neurological abnormalities, liver failure, cardiorespiratory dysfunction, gastrointestinal bleeding, low platelet count (leukocyte count ≤1000 cells/ml), pleural and pericardial effusion and ascites or death. It was a mandatory classification after 2007 [33].

Based on the results of a multicenter study (Dengue Control, DENCO) to assess the limitations of the 1997 WHO classification, experts from dengue endemic regions agreed on a binary classification represented by two clear entities, severe dengue and dengue and, the term “non-severe dengue” should be avoided, as any dengue case can become severe. Moreover, it was shown that patients exhibiting warning signs are at increased risk of severe disease progression and deserve careful observation [34]. This new classification proposed in 2009, characterized dengue infections in dengue without signs (DWoWS), DwWS and SD [1, 9, 35]. From January 2014 and on, Brazil adopted this new proposed classification. Therefore, in this analysis, DHF/DSS, DCC, DwWS and SD denominations were used, considering the epidemic year analyzed.

A higher sensitivity to detect increased disease severity has been shown by the new WHO 2009 dengue classification [36–39]. Its specificity, however, is much lower (73.0%) compared to the 1997 classification (93.4%). The higher sensitivity allows better patients’ management, reducing mortality [40, 41], on the other hand, may also result in the misclassification of some severe cases [42]. In fact, the lower specificity of this new classification is attributed, partly, to the lack of clear criteria for the definition of the warning signs [43].

DHF cases fatality rates were high in 1994, 1997, 1998, 2006, 2012, and 2013. By DCC, deaths were more frequently reported in 2003, 2006, 2007, with increasing numbers from 2008 to 2013. In 2014, 3% of DwWS patients died, while in 2014 and 2015, 8 and 7% of SD cases died, respectively. From 1986 to 1990 the DHF fatality rate was up to 10% in RJ and AL, but was five times higher in CE, from 1996 to 2001, however, from 2006 to 2010, case fatality rates increased in almost all states.

Sex has also been considered by some authors, as risk factor for the disease severity. Studies in Asia and the Americas, show that women are more likely to have the disease and are at greater risk of developing more severe forms than men [44–47]. In the 30 years period, an equal distribution of dengue fatal cases was observed between the sexes. Previous studies on dengue incidence have sometimes found equal attack rates between the sexes [48–51], and sometimes found uneven distribution of cases, with no clear tendency for males or females to be more affected [45, 52–55].

From 1986 to 2006, dengue deaths occurred more often in individuals over 15 years old. This changed in 2007–2008, with the DENV-2 re-emergence, as more than 53% of the dengue deaths cases occurred in children 15 years old and under [16]. Likewise, the study by Paixão et al. [29] analyzing dengue mortality from 2001 to 2011 in Brazil, showed the highest DHF case fatality rates on individuals over 15 years old and especially on those 80 years old and over. However, children under 1 year old experienced increased fatality rates.

After 2009, there was a decrease in fatal cases in children 15 years old and under, while fatal cases on individuals above 15 years old became more frequent, especially in the years of 2010, 2013 and 2015. The increased risk of death in the older age group may be associated with the difficulty in managing the disease in a population with a high frequency of comorbidities [56]. Cases coincident with sickle cell anemia, autoimmune diseases, asthma, hypertension, uremia and diabetes mellitus have been described in more severe outcomes of dengue [56–60].

## Conclusions

Currently, Brazil is experiencing a hyperendemic scenario, with the co-circulation of the four DENV serotypes and occurrence of severe and fatal cases and, more recently, the co-circulation with other arboviruses such as Zika, Yellow Fever and Chikungunya, Therefore, the possibility of misdiagnosis and even co-infections in a same individual and the its impact in the disease outcome, can not be neglected and need further investigation.

One point to be addressed here and pointed out in a previous study, is the challenge in determining whether a death occurs *due to* DENV infection or in a patient *with* DENV infection, meaning the disease is the cause of death or is the underlying cause of it [61]. Either way, the disease surveillance and studies characterizing what has been reported overtime, are still important tools to better understand the factors involved on the disease outcome.

It is a fact that, there are many dengue-related deaths underestimated in many health services, even after 30 years of dengue surveillance in Brazil and it has been shown that the structuring and organization of surveillance, autopsy and laboratory teams, may significantly improve this scenario [61–63].

Despite that, the use of secondary data as those analyzed here, imposes some limitations to the study and those include the lack of some clinical and/or demographic information, description of disease course during hospitalization and until death, delay in diagnosis and low adherence to notification by health professionals. Dengue cases are under-reported in Brazil and improvements are needed in the proper filing of report forms [7, 64–66].

## Abbreviations

AC: Acre
AL: Alagoas
AM: Amazonas
AP: Amapá
BA: Bahia
CE: Ceará
CE: Confidence interval
DCC: Dengue with complications
DENCO: Dengue Control
DENCO: Dengue Hemorrhagic Feverx
DENV: Dengue viruses
DF: Distrito Federal
DSS: Dengue Shock Syndrome
DwWS: Dengue with warning signs
ES: Espírito Santo
GO: Goiás
IBGE: Instituto Brasileiro de Geografia e Estatística
ICD-10: 10th International Classification of Diseases
MA: Maranhão
MG: Minas Gerais
MoH: Brazilian Ministry of Health
MS: Mato Grosso do Sul
MT: Mato Grosso
OR: Odds ratio
PA: Pará
PB: Paraíba
PE: Pernambuco
PI: Piauí
PR: Paraná
RJ: Rio de Janeiro
RN: Rio Grande do Norte
RO: Rondônia
RR: Roraima
RS: Rio Grande do Sul
SC: Santa Catarina
SD: Severe Dengue
SE: Sergipe
SIM: Mortality Information System
SINAN: Sistema de Informação de Agravos de Notificação
SP: São Paulo
TO: Tocantins
WHO: World Health Organization
